# Intraoperative immunomodulatory effects of sevoflurane versus total intravenous anesthesia with propofol in bariatric surgery (the OBESITA trial): study protocol for a randomized controlled pilot trial

**DOI:** 10.1186/s13063-019-3399-z

**Published:** 2019-05-28

**Authors:** Giselle Carvalho de Sousa, Fernanda Ferreira Cruz, Luciana Boavista Heil, Carlos José Saboya Sobrinho, Felipe Saddy, Frederico Paranhos Knibel, Joana Barreto Pereira, Marcus J. Schultz, Paolo Pelosi, Marcelo Gama de Abreu, Pedro Leme Silva, Patricia Rieken Macedo Rocco

**Affiliations:** 1 Laboratory of Pulmonary Investigation, Carlos Chagas Filho Institute of Biophysics, Federal University of Rio de Janeiro, Centro de Ciências da Saúde, Avenida Carlos Chagas Filho, 373, Bloco G1-014, Ilha do Fundão, Rio de Janeiro, 21941-902 Brazil; 2 Department of Anesthesiology, Federal University of Rio de Janeiro, Rio de Janeiro, Brazil; 3 Institute D’Or of Research and Teaching, Rio de Janeiro, Brazil; 4 Department of Intensive Care and Laboratory of Experimental Intensive Care and Anesthesiology (L·E·I·C·A), Amsterdam University Medical Centers, University of Amsterdam, Amsterdam, The Netherlands; 5 Mahidol Oxford Tropical Medicine Research Unit (MORU), Faculty of Tropical Medicine, Mahidol University, Bangkok, Thailand; 6 Department of Surgical Sciences and Integrated Diagnostics, University of Genoa, Genoa, Italy; 7 Ospedale Policlinico San Martino, IRCCS for Oncology and Neurosciences, Genoa, Italy; 8 Pulmonary Engineering Group, Department of Anesthesiology and Intensive Care Medicine, University Hospital Carl Gustav Carus, Dresden, Germany

**Keywords:** Obesity, Inflammation, Cytokines, Interleukin 6, Lung injury, Propofol, Sevoflurane, Clinical trial

## Abstract

**Background:**

Obesity is associated with a chronic systemic inflammatory process. Volatile or intravenous anesthetic agents may modulate immune function, and may do so differentially in obesity. However, no study has evaluated whether these potential immunomodulatory effects differ according to type of anesthesia in obese patients undergoing laparoscopic bariatric surgery.

**Methods/design:**

The OBESITA trial is a prospective, nonblinded, single-center, randomized, controlled clinical pilot trial. The trial will include 48 patients with a body mass index ≥ 35 kg/m^2^, scheduled for laparoscopic bariatric surgery using sleeve or a Roux-en-Y gastric bypass technique, who will be allocated 1:1 to undergo general inhalational anesthesia with sevoflurane or total intravenous anesthesia (TIVA) with propofol. The primary endpoint is the difference in plasma interleukin (IL)-6 levels when comparing the two anesthetic agents. Blood samples will be collected prior to anesthesia induction (baseline), immediately after anesthetic induction, and before endotracheal extubation. Levels of other proinflammatory and anti-inflammatory cytokines, neutrophil chemotaxis, macrophage differentiation, phagocytosis, and occurrence of intraoperative and postoperative complications will also be evaluated.

**Discussion:**

To our knowledge, this is the first randomized clinical trial designed to compare the effects of two different anesthetics on immunomodulation in obese patients undergoing laparoscopic bariatric surgery. Our hypothesis is that anesthesia with sevoflurane will result in a weaker proinflammatory response compared to anesthesia with propofol, with lower circulating levels of IL-6 and other proinflammatory mediators, and increased macrophage differentiation into the M2 phenotype in adipose tissue.

**Trial registration:**

Registro Brasileiro de Ensaios Clínicos, RBR-77kfj5. Registered on 25 July 2018.

**Electronic supplementary material:**

The online version of this article (10.1186/s13063-019-3399-z) contains supplementary material, which is available to authorized users.

## Background

Obesity, a major public health issue, has long been recognized as a precursor of morbidity and premature mortality [1]. An increasing number of morbidly obese persons - defined by the World Health Organization as having a body mass index (BMI) ≥ 40 kg/m^2^ [1] - are undergoing surgical procedures.

Obesity is a metabolic disorder associated with chronic systemic inflammation, predominantly originating from visceral adipose tissue (VAT). Adipose tissue is composed of a number of immune cells [2, 3]; among these, macrophages have been shown to be the predominant cell population [4]. In obesity, adipocyte expansion increases the secretion of monocyte chemoattractants that recruit inflammatory monocytes expressing type 2+ chemokine receptor (CCR2+) to the adipose tissue, where they differentiate into activated macrophages with the M1 phenotype [2]. M1 macrophages increase the production of proinflammatory cytokines such as tumor necrosis factor (TNF)-α, interleukin (IL)-6, and IL-12, and the generation of reactive oxygen species, such as nitric oxide (NO), via the iNOS pathway (NOS2) [2, 3].

Caution is required when choosing anesthetic agents for administration in morbidly obese patients, since these drugs may influence immune function and the inflammatory process [5]. In this context, anesthetics may interact with the perioperative inflammatory response, affecting the release of cytokines [6], the expression of cytokine receptors [7], phagocytic or cytotoxic action [8], and the transcription or translation of protein mediators [9, 10], resulting in potential beneficial or deleterious effects. Sevoflurane, a volatile anesthetic, exerts a number of effects on innate immunity, mainly inhibiting neutrophil function, decreasing lymphocyte proliferation, suppressing the release of cytokines from peripheral blood mononuclear cells and circulating monocytes, and affecting the function of natural killer (NK) and dendritic cells [8, 11, 12]. Propofol, an intravenous agent, has been shown to inhibit neutrophil chemotaxis [13], neutrophil oxidative response [14], macrophage phagocytosis [15], and bacterial clearance [16]. In a previous study, Heil et al. showed that in obese rats, anesthesia with propofol increased lung inflammation and airway resistance compared with dexmedetomidine and thiopental [17].

Previous studies compared sevoflurane with propofol on performance, effectiveness, recovery from anesthesia, and bispectral index (BIS) recordings (a marker of possible intraoperative awareness) [18, 19], but have not evaluated the immunomodulatory potential of these anesthetics in obese patients.

Overall, in centers with distinct expertise, the 30-day mortality rate in bariatric surgery is low (0.1–2%) [20], as is the rate of major complications (3.6% after gastric bypass and 2.2% after sleeve gastrectomy) [21]. However, apart from major and early complications, minor complications can still occur in the days following surgery, which may increase hospital length of stay. The inflammatory process is involved in the perioperative period, in which it impairs wound healing and increases the risk of infection. To date, no study has evaluated which anesthetic agent is associated with the least inflammation and fewest complications in obese patients undergoing surgery.

Therefore, we designed the OBESITA trial to assess the immunomodulatory effects of anesthetics and inform future sample size calculations for studies evaluating sevoflurane versus propofol in obese patients undergoing bariatric surgery. Our hypothesis is that anesthesia with sevoflurane will result in a weaker inflammatory response to surgery than anesthesia with propofol [17, 22].

## Methods

### Study design

**Fig. 1:**
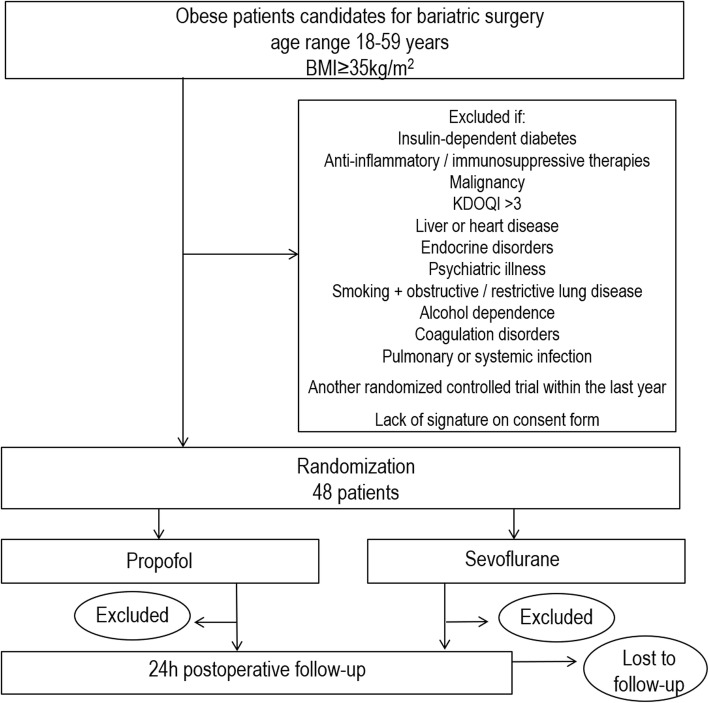
Consolidated Standards of Reporting Trials (CONSORT) diagram. BMI, body mass index, KDOQI, Kidney Disease Outcomes Quality Initiative

**Fig. 2:**
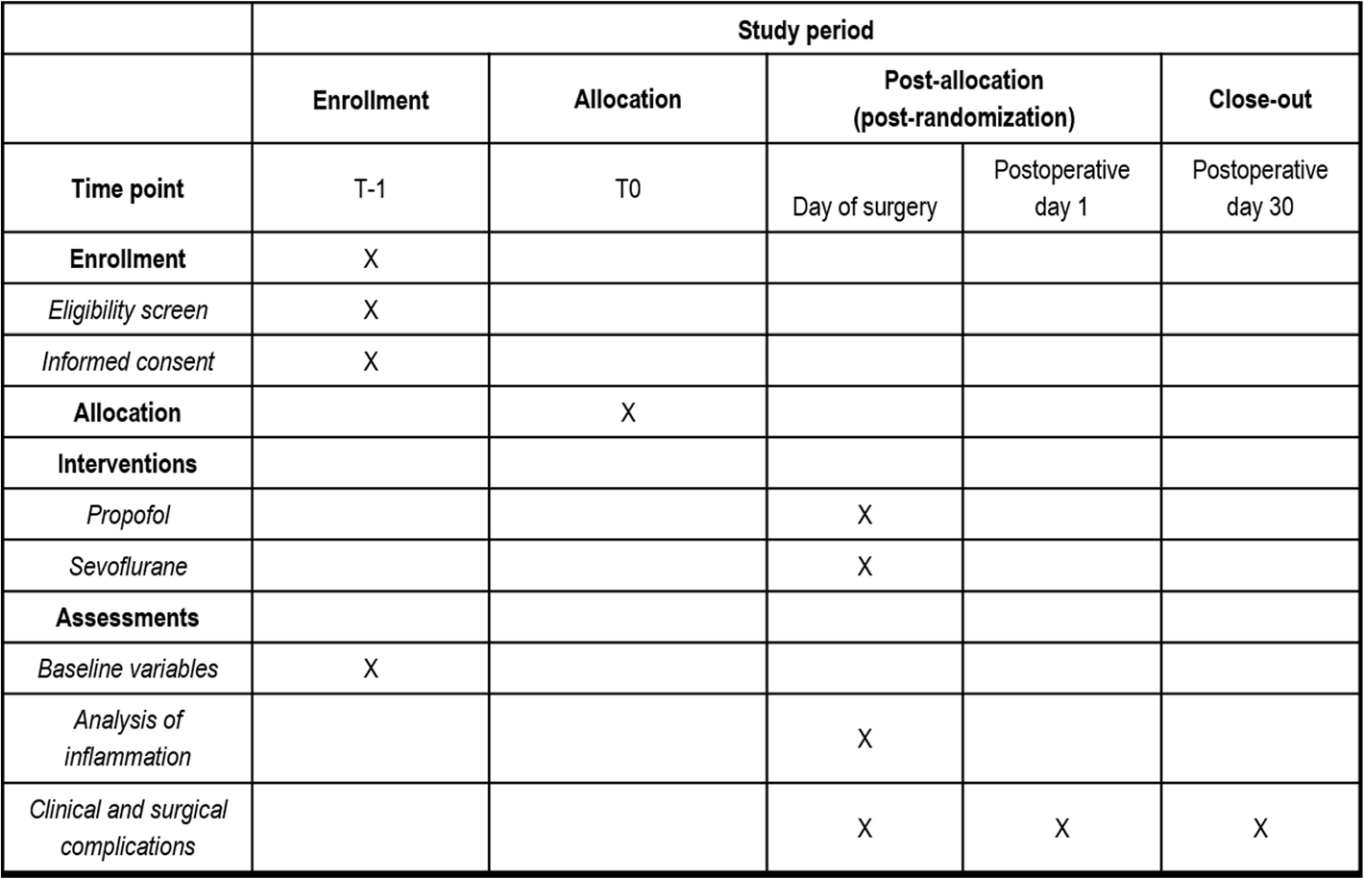
Standard Protocol Items: Recommendations for Interventional Trials (SPIRIT) diagram. Schedule of enrollment, interventions, and assessments for the OBESITA trial

The OBESITA study is a single-center, nonblinded, randomized controlled pilot trial with a 1:1 allocation ratio. A total of 48 patients scheduled to undergo laparoscopic bariatric surgery will be randomized to receive either inhalational anesthesia with sevoflurane or total intravenous anesthesia (TIVA) with propofol (see Consolidated Standards of Reporting Trials (CONSORT) diagram, Fig. 1) [23]. The protocol has been designed in accordance with the Standardized Protocol Items: Recommendations for Interventional Trials (SPIRIT) guidelines (Fig. 2) and the corresponding checklist (Additional file 1). Prior to surgery, patients will be approached by a member of the study team and informed written consent will be sought.

### Inclusion criteria

Patients aged 18–59 years with BMI ≥ 35 kg/m^2^, scheduled to undergo laparoscopic bariatric surgery under general anesthesia, will be eligible. Preoperative management of patients in both groups will be consistent with current surgical best practice and at the discretion of the operating surgeon. Patients at risk of postoperative pulmonary complications (PPCs) will be identified using the Respiratory Risk Assessment in Surgical Patients in Catalonia (ARISCAT) score [24], which consists of seven independent predictors: three patient-related and four surgery-related predictors (Table 1). An ARISCAT score ≥ 26 denotes intermediate to high risk of PPCs. All medications used by patients will be queried during the preoperative interview with the anesthesiologist.

**Table 1:** Independent predictors of risk for PPCs identified in the logistic regression model (ARISCAT score)

	Multivariate analysis OR (95% CI) *n* = 1.624*	β	Risk score^a^
Age, years			
≤ 50	1		
51–80	1.4 (0.6–3.3)	0.331	3
> 80	5.1 (1.9–13.3)	1.619	16
Preoperative SpO_2_, %			
≥ 96	1		
91–95	2.2 (1.2–4.2)	0.802	8
≤ 90	10.7 (4.1–28.1)	2.375	24
RTI in preceding month	5.5 (2.6–11.5)	1.698	17
Preoperative anemia (Hb ≤ 10 g/dL)	3.0 (1.4–6.5)	1.105	11
Surgical incision			
Peripheral	1		
Upper abdomen	4.4 (2.3–8.5)	1.480	15
Intrathoracic	11.4 (4.9–26.0)	2.431	24
Duration of surgery, hours			
≤ 2	1		
> 2–3	4.9 (2.4–10.1)	1.593	16
> 3	9.7 (4.7–19.9)	2.268	23
Emergency procedure	2.2 (1.0–4.5)	0.768	8

Three patients were excluded because of a missing value for some variables. Logistic regression model constructed with the development subsample, c-index = 0.90; Hosmer-Lemeshow chi-square test = 7.862; *P* = 0.447

*CI* confidence interval, *Hb* hemoglobin, *OR* odds ratio, *PPC* postoperative pulmonary complications, *RTI* respiratory tract infection, *SpO2* oxyhemoglobin saturation by pulse oximetry (breathing room air in the supine position)

^a^Simplified risk score was the sum of each logistic regression coefficient (β) multiplied by 10, after rounding off

### Exclusion criteria

The exclusion criteria are age < 18 years or > 59 years; insulin-dependent diabetes mellitus; anti-inflammatory and/or immunosuppressive therapies; a diagnosis of malignancy, chronic kidney disease (Kidney Disease Outcomes Quality Initiative (KDOQI) score > 3), liver disease (total serum bilirubin < 3 g/dL and total bilirubin > 5 mg/dL), or heart disease (New York Heart Association (NYHA) class III/IV); obesity caused by endocrine disorders; psychiatric illness that might interfere with capacity for formal consent and compliance; persistent smoking associated with significant obstructive lung disease (forced vital capacity (FVC) or forced expiratory volume in one second (FEV_1_) < 50% of predicted value) or restrictive lung disease (pre-bronchodilator FVC < 80% of predicted value and FEV_1_/FVC ≥ 0.7) [25]; alcohol dependence; pre-existing coagulation disorders; evident pulmonary or systemic infection (clinical diagnosis or any one of the following: C-reactive protein > 5 mg/L, leukocytosis with > 10,000 white blood cells (WBCs), or body temperature > 37 °C); autoimmune diseases; and participation in another randomized controlled trial within the preceding year.

### Intervention

Patients will be randomly assigned to receive general anesthesia with either sevoflurane alone or propofol alone. Anesthesia will be maintained with approximately 1 minimum alveolar concentration (MAC) in the sevoflurane group; in the propofol group, anesthesia will be maintained with a continuous infusion of 2–4 μg/mL in a target- controlled infusion pump programmed with the Marsh pharmacokinetic model, using adjusted body weight, aiming to maintain a suitable level of hypnosis (bispectral index (BIS) 40–60) [26].

### Minimization of bias

Patients will be randomized using a computer-generated random number table, with an allocation ratio of 1:1. The allocation sequence will be implemented by a telephone call at the time of surgery. An investigator will collect data, and blood and adipose tissue samples will be coded so that the investigator evaluating the laboratory outcomes remains blinded to group allocation.

### Standard care

Patients assigned to both groups will receive intravenous fluids at a volume of 10–15 mL/kg total body weight (TBW) if possible [27, 28]. According to patient randomization, anesthesia in the TIVA with propofol group will be induced using propofol (1.5–2 mg/kg ideal body weight (calculated as height in cm – x, where x = 100 in adult men and 105 in adult women)) [29], whereas in the sevoflurane group, anesthesia will be induced using midazolam (0.05–0.15 mg/kg TBW) [29]. All patients will then receive alfentanil (15–20 μg/kg lean body weight (LBW), defined in men as 1.1 × (weight) − 128 × (weight / height)^2^ and in women as 1.07 × (weight) – 148 × (weight/height)^2^ [30] and rocuronium (1.2 mg/kg IBW)) [31]. Patients will receive a continuous infusion of remifentanil (0.1–0.4 μg/kg/min LBW) after induction of anesthesia and during surgery [31]. All patients will undergo neuromuscular monitoring with quantitative devices [32]. Patients will be extubated using sugammadex 2 mg/kg IBW if the train of four (TOF) is ≥ 2 responses or sugammadex 4 mg/kg IBW if TOF = 0 and the post-tetanic count (PTC) is ≥ 1 (33) [33].

After closure of the surgical incision, morphine (0.08 mg/kg IBW) will be administered intravenously [29]. Postoperative analgesia will consist of dipyrone (metamizole) 1 g every 6 h, ketoprofen 100 mg every 12 h or parecoxib 40 mg every 12 h, and tramadol 50–100 mg every 8 h or as rescue analgesia. Ondansetron (4 mg every 8 h) will be administered as prophylaxis for nausea and vomiting.

### Samples

Blood (15 mL) will be collected in heparinized tubes prior to induction of anesthesia (baseline), immediately after anesthetic induction (T1), and before extubation (T2).

Two small fragments of subcutaneous adipose tissue and visceral adipose tissue will also be sampled. Each fragment will measure 2 × 2 × 2 cm after excess blood vessels and connective tissue have been removed with the aid of a scalpel, and will be obtained from surgical specimens that would be discarded immediately following gastroplasty.

### Mechanical ventilation

Patients from both groups will be mechanically ventilated in pressure-controlled mode, with a tidal volume (V_T_) of 5–7 ml/kg IBW, respiratory rate (RR) adjusted to maintain arterial partial pressure of carbon dioxide (PaCO_2_) between 36 and 44 mmHg, and a positive end-expiratory pressure (PEEP) of 5 cmH_2_O [34]. The inspired fraction of oxygen (FiO_2_) will be adjusted to maintain oxygen saturation of hemoglobin (SaO_2_) > 95% (0.4–0.5).

### Rescue strategies for intraoperative hypoxemia

Based on the protocol used in the PROBESE trial [35], if oxygen saturation falls to 92% or below, factors such as increased airway resistance, presence of intrinsic PEEP, hemodynamic instability, and ventilator malfunction should be excluded.

Initially, FiO_2_ should be raised gradually. Elevation of PEEP is restricted to more severe cases, when hypoxemia persists with FiO_2_ = 1.0. In this case, PEEP is gradually increased to 7 cmH_2_O. Alveolar recruitment maneuvers should be performed if SaO_2_ remains ≤ 92% [35].

Anesthesiologists may deviate from the ventilation protocol at any time if concerns about patient safety arise. PEEP may be modified at the anesthesiologist’s discretion.

### Measurements

The following ventilatory parameters will be measured at three time points - after anesthetic induction (approximately 10 min), after induction of pneumoperitoneum (approximately 20 min), and at the end of surgery: minute ventilation, RR, V_T_, peak and plateau pressures, PEEP, static and dynamic compliance, FiO_2_, and SaO_2_.

Arterial blood gases and hemoglobin level will be measured immediately after induction of anesthesia and before extubation.

Heart rate (HR) and noninvasive mean blood pressure (NIBP) will be measured throughout the procedure. The duration of surgery, the time before and after pneumoperitoneum, intra-abdominal pressure throughout the procedure, and the volume insufflated to achieve pneumoperitoneum will also be recorded.

### Postoperative data

The following clinical data will be evaluated within 24 h of surgery: vital signs (HR, NIBP, RR, and axillary temperature (AT)), complete physical examination, presence of organ dysfunction, visual analogue scale (VAS) score for pain, and postoperative complications (pulmonary infection, systemic inflammatory response syndrome, sepsis, septic shock, extrapulmonary infection, coma, acute myocardial infarction, acute renal failure, disseminated intravascular coagulation (DIC), gastrointestinal failure (GIF), and acute liver failure).

### In vitro assays

#### Blood samples

Blood samples will be collected from all patients at baseline, T1, and T2. Heparinized tubes will be immediately centrifuged at 1500 × *g* for 5 min at 4 °C for separation of plasma and cells. These samples will be centrifuged within 6 h of collection. Plasma will be stored at − 80 °C until the time of analysis. The cell pellet will be used for monocyte and neutrophil isolation and evaluation, while plasma will be used later (after perioperative monitoring) for quantification of C-reactive protein (CRP), procalcitonin (PCT), IL-6, IL-10, and TNF-α levels.

#### Adipose tissue fragments

Adipose tissue fragments will be subjected to enzymatic digestion with 1 mg/mL collagenase IA (Sigma Chemical Co., St. Louis, USA) for 30 min at 37 °C. The remaining tissue will be filtered through a 100-mm pore nylon mesh, and proteolysis will be interrupted with high-glucose Dulbecco’s modified Eagle’s medium (DMEM, Gibco, Invitrogen, NY, USA) with 20% fetal bovine serum (FBS) (Hyclone Laboratories Inc., UT, USA) and 1% penicillin and streptomycin solution (Gibco, Invitrogen, NY, USA). The cells will be centrifuged and washed twice with 0.01 mM phosphate-buffered saline (PBS), and will then be prepared for magnetic selection.

Total leukocytes obtained from blood and adipose tissue will be analyzed by diluting the samples in Türk’s liquid (1:10) and counting cells in a Neubauer chamber under a light microscope (BX51, Olympus, Tokyo, Japan). Slides will be stained for the differential cell count using the May- Grünwald-Giemsa method and visualized with an oil- immersion objective under the same microscope. Monocytes and neutrophils will be isolated by the magnetic sorting technique (Invitrogen, CA, USA) through incubation with biotin-associated anti-CD11b and anti-CD11c antibodies, respectively, followed by incorporation with biotin- coated iron beads and exposure to a magnetic field for 10 min.

Total messenger RNA (mRNA) will be extracted from cells using the Reliaprep RNA Tissue Miniprep System RNA extraction kit (Promega, Madison, WI, USA), following the manufacturer’s recommendations. After extraction, the precipitated RNA will be solubilized in 20 μL of diethylpyrocarbonate (DEPC)-treated water. The total RNA concentration will be measured by spectrophotometry in a Nanodrop ND-1000 system. The integrity of the samples will be verified by 1% agarose gel electrophoresis containing 0.5 μg/mL of ethidium bromide. First-strand complementary DNA (cDNA) will be obtained from total RNA using the High Capacity cDNA Reverse Transcription kit (Invitrogen, CA, USA). Relative mRNA levels will be measured using BRYT Green (Promega, Fitchburg, WI, USA) using a Mastercycler® ep Realplex PCR system (Eppendorf, Hamburg, Germany). All experiments will be performed in triplicates. Growth factors and inflammatory mediators will be analyzed by real-time RT-PCR. The relative level of each gene will be normalized to the housekeeping gene acidic ribosomal phosphoprotein P0 (*36B4*) and expressed as fold change relative to each individual at initial conditions, using the 2^−ΔΔ^ cycle threshold (Ct) method, where ΔCt = Ct (target gene) − Ct (housekeeping gene). Neutrophil and macrophage gene expression in vitro will be analyzed by an investigator blinded to group assignment.

### Study endpoints

#### Primary

The primary endpoint is the difference in plasma levels of IL-6 when comparing patients anesthetized with sevoflurane versus propofol.

#### Secondary

The secondary endpoints are (1) the differences in levels of other proinflammmatory and anti-inflammatory mediators in blood, subcutaneous tissue, and visceral adipose tissue, namely (a) inflammatory mediators associated with neutrophils: metalloprotease (MMP)-2 [36–38], MMP-9 [36], CXCR2 [39], IL-12 [40], CCL3 (macrophage inflammatory protein (MIP)-1α) [41–44], CCL2 (MCP1) [44], IL-1β [45], TNF-α [46], NRF2 [47, 48], (b) mediators associated with macrophages: proinflammatory (IL-1β, CD40 [49], CD80 [50]), M1 phenotype markers (TNF-α, IL-6 [51–53], iNOS [54–56]), anti-inflammatory (IL-1 receptor antagonist (IL-1RA) [57–59], NrF2), M2 phenotype markers (CD163 [60, 61], CD206 [62], arginase [63, 64], IL-10 [65], transforming growth factor (TGF)-β1 [66–68]), and (c) mediators associated with cell death pathways (BCL2-associated X (Bax), bcl2 [69, 70], caspases 3 and 9 [71–73]); (2) intraoperative complications: (a) hypoxemia (PaO_2_/FiO_2_ ratio < 300), (b) hypotension (systolic blood pressure < 90 mmHg), (c) bradycardia (heart rate < 50 bpm), (d) unexpected need for intensive care unit (ICU) admission or readmission, (e) length of hospital stay, (f) number of days at home in the first month after surgery, and (g) postoperative complications: pulmonary infection (defined as new or progressive radiographic infiltrate and at least two of the following criteria - antibiotic treatment, axillary temperature > 38 °C, leukopenia (WBC < 4000 cells/mm^3^) or leukocytosis (WBC > 12,000 cells/mm^3^), and/or purulent secretions); sepsis (defined as suspicion or certainty of infection and an acute increase ≥ 2 points in the Sequential Organ Failure Assessment Score (SOFA) in response to an infection, representing organ dysfunction); septic shock (defined as sepsis plus need for vasopressor to maintain mean blood pressure > 65 mmHg plus lactate > 2 mmol/L [18 mg/dL] after adequate volume resuscitation [74–76]); extrapulmonary infection (of the operative wound or any other infection); coma (Glasgow Coma Scale < 8 in the absence of therapeutic sedation); acute myocardial infarction (according to the universal definition thereof [77]); acute kidney failure (according to the risk, injury, failure, loss, end-stage renal failure (RIFLE) criteria classification system [78]); DIC (according to the International System for the Evaluation of Thrombosis and Hemostasis for DIC [79]); GIF (defined according to the GIF score [80]); acute liver failure (defined as a ratio of total bilirubin on the 5th postoperative day to the 1st postoperative day > 1.7 plus a ratio of international normalized ratio (INR) on the 5th postoperative day to the 1st postoperative day > 1.0 or presence of hepatic encephalopathy and INR > 1.5) [81, 82].

## Statistical analysis

Positive modulation of the IL-6 receptor and IL-6 expression in adipose tissue is known to occur in obesity [51, 83]. A convenience sample of 48 patients has been chosen on the basis of a study in which plasma IL-6 levels were compared before and after anesthesia in patients undergoing thoracic surgery with one-lung ventilation [84]. The sample size was calculated using G*Power 3.1.9.2 software (University of Dusseldorf, Germany), taking into account an effect size of 1.33, a two-sided test, and a ratio of 1. A sample size of 10 patients in each group will provide adequate power (80%), assuming α = 0.05, to detect at least an effect size of 33% difference in IL-6 levels in plasma samples collected immediately after induction of anesthesia and before extubation in obese patients anesthetized with sevoflurane or propofol (*p* = 0.05). Considering possible losses and to obtain even more significant results, we chose to increase the sample to 24 patients per group. Data will be tested for normality and variability. Comparison of the parameters of interest between participants in the sevoflurane and propofol groups will be performed by Student’s *t* test or the Wilcoxon test, as appropriate. Statistical significance will be accepted at *p* < 0.05. All analyses will be undertaken by an independent statistician, who will be blinded to group allocation.

## Discussion

The worldwide epidemic of obesity has become a major health problem. Obesity is known to feature a chronic inflammatory process, with increased levels of proinflammatory cytokines (TNF-α, IL-6, IL-12), generation of reactive oxygen species, and greater differentiation of activated macrophages into the M1 phenotype [2, 3]. Macrophages are also associated with the development of insulin resistance and diabetes mellitus [85, 86]. Neutrophils are important components of the acute inflammatory response, constituting the first line of innate defense against infectious diseases. They also play a role in immune-response regulation [87], recruitment of macrophages and dendritic cells, and clearance of debris by means of phagocytosis [87, 88]. In addition, they release antimicrobial molecules and neutrophil extracellular traps (NETs), which degrade bacterial virulence factors and kill extracellular bacteria, binding to both Gram-negative and Gram-positive bacteria [87, 89].

As the obese population increases, so does the need for diagnostic or therapeutic interventions where anesthetic care and management of anesthetic agents are required. It is well-established that anesthesia and surgery provoke hemodynamic, metabolic, and inflammatory responses, even though the isolated effects of each cannot be easily understood. Understanding the immunomodulatory properties of anesthetics in the obese body would be very helpful in establishing better guidelines for anesthetic management of this population.

Immunomodulation may have a beneficial effect, since suppression of the exacerbated inflammatory response can avoid damage such as that caused by acute inflammation [90], or a deleterious effect, since it can prevent the body from neutralizing infectious pathogens. It is now known that anesthetic agents may compromise or enhance immune function [5].

There are several patient-related (and thus nonmodifiable) factors associated with increased incidence of postoperative complications after bariatric surgery: male sex, preoperative BMI ≥ 60 kg/m^2^, open surgery, smoking within the last year, deep vein thrombosis, therapeutic anticoagulation, and serum albumin < 3.5 g/dL [91]. The duration and invasiveness of the surgical procedure may affect the inflammatory response, thus increasing the risk of bias [91], and will therefore be taken into account in this trial; these factors will be individually analyzed post hoc to isolate their interference with the outcomes of interest. The role of positive end-expiratory pressure (PEEP) in mechanical ventilation during general anesthesia for surgery remains uncertain. An ongoing randomized clinical trial is testing two different levels of PEEP: 4 cmH_2_O without recruitment maneuvers versus 12 cmH_2_O with recruitment maneuvers [35, 92]. In the present trial protocol, a PEEP of 5 cmH_2_O is suggested based on a secondary analysis of the international multicenter LAS VEGAS study, restricted to obese patients [93].

To our knowledge, only one prospective study has compared the effect of anesthesia with sevoflurane on inflammation to that of TIVA with propofol, in 54 adults undergoing thoracic surgery with one-lung ventilation [22]. The authors observed that the increase in inflammatory mediators such as TNF-α, IL-6, and monocyte chemoattractant protein 1 (MCP-1) was significantly blunted in the sevoflurane group. Sevoflurane was also associated with fewer postoperative adverse events [22]. A randomized controlled trial of 5400 patients, which compared volatile anesthetics to TIVA for elective cardiac surgery, showed that anesthesia with a volatile agent did not result in a significantly lower 1-year mortality rate compared to TIVA [94].

Moreover, in the experimental setting of obesity, a 1-h infusion of propofol resulted in increased airway resistance, atelectasis, and pulmonary inflammation mediated by increased TNF-α and IL-6 in lung tissue, with depletion of antioxidative enzymes [17]. Based on this evidence, we hypothesize that anesthesia with sevoflurane will result in a weaker inflammatory response compared to anesthesia with propofol.

This study is the first step toward choosing an anesthetic strategy that is capable of reducing systemic inflammation in obese subjects (i.e., patients with chronic inflammation) undergoing laparoscopic bariatric surgery. Some studies have compared sevoflurane with propofol, but have focused on clinical consequences, such as intraoperative mean arterial pressure, eye opening, extubation, recovery from anesthesia, postoperative pain, and incidence and severity of postoperative nausea and vomiting (PONV) [18, 95], without understanding the mechanisms associated with clinical improvement.

Sevoflurane, a low-solubility inhaled anesthetic, is the first-line anesthetic agent of choice in Brazil. It has been shown to have a cardioprotective effect, preventing myocardial ischemia and arrhythmias [96], even though like other anesthetic agents it may cause hypotension [97]. A study determined that the minimal alveolar concentration (MAC) for sevoflurane needed to maintain the BIS at < 50 in morbidly obese patients undergoing bariatric surgery [98] was 1.8–1.6%, higher than those values previously reported in normal adult patients (0.97%; 95% CI 0.89–1.1%) and lower than those reported in children (2.8%; 95% CI 2.7–3.1%), which justifies the minimal MAC of 1.0 defined for this protocol. Moreover, a double-blind randomized controlled trial in superobese patients (BMI > 50 kg/m^2^), in which the performance, effectiveness, and recovery from anesthesia was compared for sevoflurane versus propofol in combination with remifentanil, showed that although both propofol and sevoflurane provided adequate general anesthesia, sevoflurane may be preferable in the superobese due to superior hemodynamic stability and faster recovery [18].

Our analysis of biomarkers of obesity-related inflammation will help to further characterize the influence of anesthetics on the inflammatory response in obese subjects undergoing laparoscopic bariatric surgery. Potentially relevant biomarkers related to neutrophils (MMP-2, MMP-9, CXCR2, IL-12, CCL3, CCL2, IL-1β, TNF-α, NRF2), macrophages (IL-6, IL-1β, TNF-α, iNOS, CD40, CD80, CD163, CD206, arginase, IL-10, IL-1RA, TGF-β1, NrF2), and cell death pathways (bax, bcl2, caspase 3, caspase 9) will be evaluated. Table 2 summarizes the main roles of these biomarkers and their potential relevance. This study will provide a better understanding of the molecular mechanisms involved in the interaction between anesthetics and the inflammatory response, including the differential polarization of monocytes/macrophages in obese patients undergoing surgery.

**Table 2:** Biomarkers related to obesity and their relevance in the inflammatory response

Biomarkers	Relevance	References
MMP-2	Ability to break down ECM. Potential role as activator or inhibitor in tissue remodeling, atherosclerosis, cardiovascular diseases, and obesity. Levels are increased in obesity	[36–38]
MMP-9	Ability to break down ECM. Potential role as activator or inhibitor in tissue remodeling, cardiovascular diseases, and obesity. Levels are diminished in obesity	[36]
CXCR2	Expressed on circulating neutrophils; critical for directing their migration to inflammatory sites	[39]
IL-12	Associated with insulin resistance. Divergently regulated in relation to inflammatory stress, excessive energy intake, and genetic obesity	[40]
CCL3 (MIP-1α)	High transcript and protein levels in the white adipose tissue of the obese. Correlated with fasting plasma insulin concentrations in humans. Required for macrophage infiltration in adipose tissue with CCL2	[41–44]
CCL2 (MCP1)	Required for macrophage infiltration of adipose tissue Adipose-tissue and serum CCL2 expression is increased through insulin stimuli, more in insulin-resistant than in insulin-sensitive lean mice	[44]
IL-1β	Produced by macrophages. Implicated in the development of obesity-associated insulin resistance through inhibition of insulin signal transduction	[45]
TNF-α	Expressed and secreted by adipose tissue. Levels associated with degree of adiposity and insulin resistance. Targeting TNF-α and/or its receptors has been suggested as a promising treatment for insulin resistance and type 2 diabetes mellitus	[46]
Nrf2	Involved in resistance to oxidative stress. Functions as a xenobiotic-activated receptor (XAR) to regulate the adaptive response to oxidants	[47, 48]
IL-6	Pleiotropic cytokine. A central player in the regulation of inflammation, hematopoiesis, immune responses, and host defense mechanisms. Influences secretion of adipokines from adipocytes. Involved in the etiology of obesity-related comorbidities, including insulin resistance and accelerated atherosclerosis, in humans	[51–53]
iNOS	Synthesizes large quantities of nitric oxide (NO), which acts with reactive oxidative species to producing nitrosative stress, thus playing a key role in adipocyte function and glucose tolerance	[54–56]
CD 40	Ameliorates inflammation in visceral adipose tissue. Attenuates obesity-induced insulin resistance	[49]
CD 80	Plays a homeostatic role in preventing adipose inflammation	[50]
ICAM-1	Levels increased in obesity. Positively correlated with central adiposity and insulin resistance	[50, 51]
CD 163	Marker of macrophages with anti-inflammatory properties. Increased basal CD163 levels are related to obesity and its metabolic complications	[60, 61]
CD 206	Marker of M2-like macrophages in adipose tissues. Inhibits growth and differentiation of adipocyte progenitors, thus controlling adiposity and systemic insulin sensitivity	[62]
Arginase	Marker of M2 macrophages. Also expressed in endothelial cells. Involved in obesity-induced vascular dysfunction	[63, 64]
IL-10	Anti-inflammatory cytokine. High levels found in obese women. Low levels are associated with the metabolic syndrome	[65]
IL-1RA	Indirectly elicits an anti-inflammatory response. Competitively binds to the IL-1 receptor on the cell surface, thereby inhibiting the inflammatory effects of IL-1	[57–59]
TGF-β1	Anti-inflammatory cytokine. Counteracts the effects of the pro-inflammatory cytokines such as IL-8. Inhibits differentiation of pre-adipocytes	[66–68]
Bax, Bcl-2	Part of the Bcl-2 family of proteins, which constitute a cell-death pathway. Bcl-2 is a death antagonist, and Bax, a death agonist. The setpoint that determines cell susceptibility to apoptosis is determined by the ratio of these molecules. Related to brown adipose tissue atrophy (BAT) in obesity, in part due to apoptosis of adipocytes	[69, 70]
Caspase 3 and Caspase 9	Mediate the inflammatory response and apoptotic cell death to maintain homeostasis. Caspase-dependent apoptosis is involved in the pathogenesis of obesity and progression of severe nonalcoholic steatohepatitis (NASH). Caspase 9 is an initiator and caspase 3 is an executioner of cell death	[71–73]

*MMP-2* metalloproteinase-2, *ECM* extracellular matrix, *MMP-9* metalloproteinase-9, *CXCR2* CXC chemokine receptor 2, *IL-12* interleukin-12, *CCL3* C–C motif chemokine ligand 3, *MIP-1* macrophage inflammatory protein, *CCL2* C–C motif chemokine ligand 2, *MCP1* monocyte chemoattractant protein-1, *IL-1β* interleukin-1β, *TNF-α* tumor necrosis factor alpha, *Nrf2* nuclear factor (erythroid-derived 2)-like 2, *IL-6* interleukin-6, *iNOS* inducible nitric oxide synthase, *CD 40* cluster of differentiation 40, *CD 80* cluster of differentiation 80, *ICAM-1* intercellular adhesion molecule 1, *CD 163* cluster of differentiation 163, *CD 206* cluster of differentiation 206, *IL-10* interleukin-10, *IL-1RA* interleukin 1 receptor antagonist, *TGF-β1* transforming growth factor beta 1, *Bax* BCL2-associated X, *Bcl-2* B-cell lymphoma 2

Although several studies [17, 26, 33, 99–104] have been conducted in the search for an ideal drug combination or anesthetic strategy for the obese population, no guidelines have been established for laparoscopic bariatric surgery, a complex procedure that has several perioperative and postoperative repercussions. Many of these complications are due in part to the inflammatory stimulus associated with obesity. Knowledge of the immunomodulatory profile of some commonly used anesthetic agents in the obese population undergoing laparoscopic bariatric surgery may improve perioperative management, possibly reducing surgical complications such as anastomosis dehiscence, airway hyperreactivity, respiratory infection, surgical wound infection, myocardial injury, and prolonged hospitalization, with the potential for impact on short-term and long-term outcomes.

One limitation of the current trial is that, due to the nature of the intervention, blinding is not possible during surgery; this could induce bias. Nevertheless, the primary endpoint of the trial is a laboratory value and all analyses will be carried out in blinded fashion. In addition, the protocol strictly controls all processes that may influence the primary outcome. The second limitation is the choice of agents used for induction of anesthesia (alfentanil, remifentanil, and midazolam), all of which may affect the inflammatory process [8, 105, 106]. Therefore, we will strive whenever possible to minimize the interference of these anesthetics - for instance, by standardizing the use of opioids in both groups. Midazolam may not be the first-line induction agent of choice for morbidly obese patients, since its half-life is only 6 to 15 min [107]; however, hypnotic agent options for anesthetic induction in the sevoflurane group in this trial are extremely limited, since many of these agents (ketamine, dexmedetomidine, propofol) may also affect host immunomodulation [8], while systemic clearance of midazolam is unchanged in these patients [108]. Additionally, to avoid overlapping effects, we cannot use propofol in both groups. Due to ethical reasons, we are unable to use propofol alone as the anesthetic agent for bariatric surgery, since its analgesic properties are considered insufficient. The use of sevoflurane alone in morbidly obese patients is also not recommended; since these patients must be ventilated prior to intubation, the stomach may fill with air if an inhaled agent is used for induction, thus increasing the risk of regurgitation and aspiration of gastric content. Therefore, rapid sequence induction (which consists of the intravenous administration in rapid succession of both a quick-onset anesthetic induction agent and a fast-acting muscle relaxant) has been recommended in this setting [109].

The opioid dose proposed in the protocol may be an additional point of concern. Opioid-free analgesia is increasingly popular and has numerous advantages, including lower healthcare resource utilization, lower postoperative opioid requirements, and lower rates of postoperative nausea and vomiting [110, 111]. However, opioid-free narcosis with ketamine or dexmedetomidine would interfere with our results and potentially lead to confounding and bias, since these drugs also have immunomodulatory properties, as noted previously.

Finally, we would like to include patients with a wider range of clinically relevant comorbidities which are highly prevalent in the obese population, including insulin-dependent diabetes mellitus, chronic kidney disease requiring renal replacement therapy, and cardiac impairment (NYHA III-IV). Unfortunately, inclusion of these patients could also result in bias, as these conditions are all associated with greater systemic inflammation than is found in otherwise “healthy” morbidly obese patients (i.e., those without comorbidities) [112–115].

This is not a conclusive trial; it is a pilot study that aims to compile evidence of whether a further, larger study powered for hard-outcome assessment would be feasible in obese patients undergoing bariatric surgery. The upcoming results of this trial would enable the authors to prepare a larger, well-designed parallel study assessing the influence of both anesthetics on core clinical endpoints, such as mortality and morbidity, and further analyses of the immunological factors of each anesthetic once clinical endpoints are determined.

In conclusion, this randomized controlled pilot trial aims to test the hypothesis that anesthesia with sevoflurane compared to propofol in obese patients undergoing laparoscopic bariatric surgery will promote a weaker inflammatory response, as reflected by levels of IL-6 and several other parameters. This is the first study to evaluate the impact of two widely used anesthetics on the inflammatory response and their immunomodulatory properties, in addition to clinical, hemodynamic, and ventilatory outcomes, in obese patients undergoing laparoscopic bariatric surgery. Whatever the results of the trial, performing a prospective, randomized study in this setting could provide scientific evidence on the immune effects of these commonly used anesthetic agents in the obese population.

## Additional file

Additional file 1: Standard Protocol Items: Recommendations for Interventional Trials (SPIRIT) 2013 Checklist: recommended items to address in a clinical trial protocol and related documents. (DOC 124 kb)
